# Transiently elevated plasma methionine, *S*‐adenosylmethionine and *S*‐adenosylhomocysteine: Unreported laboratory findings in a patient with NGLY1 deficiency, a congenital disorder of deglycosylation

**DOI:** 10.1002/jmd2.12064

**Published:** 2019-07-22

**Authors:** Caitlin A. Chang, Xing‐Chang Wei, Steven R. Martin, David S. Sinasac, Walla Al‐Hertani

**Affiliations:** ^1^ Department of Medical Genetics Cumming School of Medicine, University of Calgary Calgary Alberta Canada; ^2^ Department of Radiology Cumming School of Medicine, University of Calgary Calgary Alberta Canada; ^3^ Department of Pediatrics Cumming School of Medicine, University of Calgary Calgary Alberta Canada; ^4^ Alberta Children's Hospital Research Institute University of Calgary Calgary Alberta Canada; ^5^ Division of Genetics and Genomics, Department of Pediatrics Boston Children's Hospital Boston Massachusetts

**Keywords:** disorders of deglycosylation, methionine, NGLY1, SAH, SAM

## Abstract

We report on a 5‐year‐old female born to consanguineous parents, ascertained at the age of 23 months for an elevated plasma methionine level, a mildly abnormal total plasma homocysteine (tHcy), and elevated aminotransferases. She had global developmental delay, microcephaly, dysmorphic facial features, hypotonia, nystagmus and tremor in her upper extremities. Metabolic investigations demonstrated elevations in plasma methionine, plasma *S*‐adenosylmethionine (SAM) and plasma *S*‐adenosylhomocysteine (SAH), with normal urine adenosine levels. Some of the elevations persisted for over 1 year. Sequencing of the *ADK* and *AHCY* genes was negative for causative variants. Plasma methionine normalized 1 year after ascertainment, but SAM and SAH continued to be elevated for six more months before normalization, and aminotransferases remained mildly elevated. Whole exome sequencing demonstrated a homozygous pathogenic variant; NM_018297.3(NGLY1):c.1405C>T (p.Arg469*) in exon 9 of the *NGLY1* gene, for which both parents were heterozygous. To our knowledge, this is the first report of NGLY1 deficiency with elevations in plasma methionine, SAM and SAH and a slight elevation of tHcy. Less than 20 patients have been reported with NGLY1 deficiency worldwide and this case expands on the biochemical phenotype of this newly discovered inborn error of metabolism.

## 1 INTRODUCTION

N‐glycanase 1 (NGLY1) deficiency was first reported in 2012,^1^ and is the first described congenital disorder of deglycosylation (NGLY1‐CDDG).^2^ It is inherited in an autosomal recessive manner, and results in multi‐system involvement with dysmorphic features, acquired microcephaly, severe hypotonia, seizures, poor growth, global developmental delay, movement disorder, and alacrima/hypolacrima.

Herein we describe a patient found to be homozygous for a pathogenic variant NM_018297.3(NGLY1):c.1405C>T (p.Arg469*) in the *NGLY1* gene. Her features included global developmental delay, microcephaly, dysmorphic facial features, hypotonia, hypolacrima, and tremor in the upper extremities. Novel findings included initially persistent, but resolving elevations in plasma methionine, plasma *S*‐adenosylmethionine (SAM) and plasma *S*‐adenosylhomocysteine (SAH), but with normal urine adenosine levels. To our knowledge, fewer than 20 patients have been reported with NGLY1 deficiency worldwide. Our patient highlights an unreported presentation of NGLY1 deficiency and suggests possible additional pathways for investigation.

## 2 PATIENT REPORT

The patient was born to a healthy 30‐year‐old Gravida 3 Para 2 mother at 32^+2^ weeks gestation, via Caesarian section, for decreased fetal heart rate. Birthweight was 1255 g (10th percentile), length was 38 cm (10th percentile), and head circumference was 28.5 cm (45th percentile). Her parents are of Libyan descent and first cousins. Both parents were healthy, as were the proband's two older male siblings. The patient required an 8‐week stay in the NICU for management of feeding and growth difficulties. Aminotransferases in the newborn period were normal.

Elevated aminotransferases were first noted at 23 months of age as part of a workup for failure to thrive; AST of 358 U/L (normal: 10‐45 U/L) and ALT of 853 U/L (normal: 1‐35 U/L) and GGT of 101 U/L (normal: 8‐35 U/L). Alpha Fetoprotein was within normal limits for her age. An initial search for alpha‐1 antitrypsin deficiency, cystic fibrosis, infectious and autoimmune etiologies was negative, and a liver ultrasound for structural changes was normal. Liver biopsy was not performed. At 3 years, the GGT normalized but aminotransferases remained elevated, with AST 216 U/L at 3 years and 58 U/L at 5 years of age, and ALT 453 U/L at 3 years and 57 U/L at 5 years of age.

**Figure 1:**
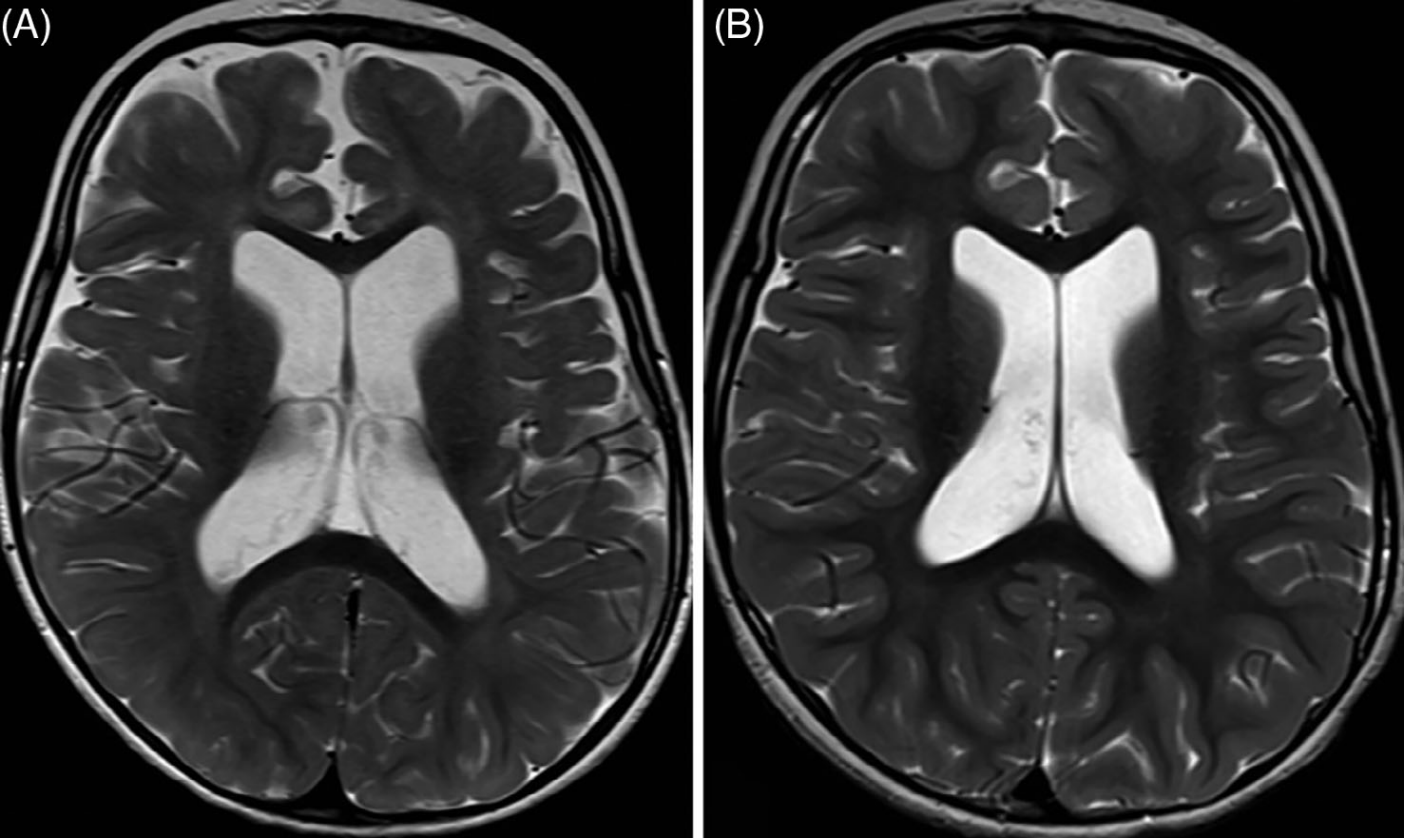
Brain MRI. T2‐weighted images at age of 24 months (A) and 5 years (B) show lateral ventricles and cerebral sulci that are mildly prominent for age

A brain MRI at 2 years of age demonstrated age‐appropriate myelination with mildly prominent cerebral sulci, lateral and third ventricles, consistent with cerebral atrophy (Figure 1A). There were no documented seizures. She had a longstanding history of tremor in the upper extremities bilaterally, as well as weakness in her legs. She was followed by ophthalmology for myopic astigmatism, esotropia, and hypolacrimation, with normal dilated fundus examination. Hearing was normal. At 5 years of age, brain MRI demonstrated persistent mildly prominent cerebral sulci, lateral, and third ventricles (Figure 1B). ^1^H‐magnetic resonance spectroscopy was normal.

An echocardiogram performed at 1 month of age demonstrated small atrial septal defect, patent ductus arteriosus, and mild right and left ventricular hypertrophy, with normalization on repeat. Electrocardiogram was normal. Complete blood counts and coagulation studies, albumin, immunoglobins, and thyroid studies were normal. There was no history of lactic acidosis. She had two fractures of the left lower extremity, after trauma. A bone age performed at 4 years; 4 months was within normal limits. Bone mineral density was reported as low, with a lumbar *Z* score of −2.1 (0.379 g/cm^2^). She was small for age, with measurements consistently at or just above the third percentile for height, weight, and head circumference.

At 5 years of age, development was delayed globally. She was unable to walk unassisted until 4 years of age. She was able to walk up and down stairs with assistance. She had between 15 and 20 words. She was able to follow two‐step commands, and primarily communicated by taking one of her parents' hands or pointing. Fine motor development was delayed, but she was able to finger feed and hold a cup and a pencil with a fisted grasp. Social development was notable for social anxiety, but she demonstrated good eye contact and a social smile. She was not yet toilet trained.

On examination at 5 years of age, her weight was 18.9 kg (50th percentile), height 107 cm (25th percentile), and head circumference 48 cm (3rd percentile), consistent with microcephaly. She had dysmorphic facial features including deep‐set eyes with long eyelashes, short palpebral fissures, and synophrys with full, arched eyebrows. Extremities were remarkable for bilateral 5th finger clinodactyly and fetal pads on all fingers. Cardiac, respiratory, and abdominal examination was unremarkable. There was no scoliosis. On neurological exam, she had increased tone with slight catching in the upper extremities bilaterally, and peripheral tremor in her upper extremities. Tone in the lower extremities was normal. She had decreased deep tendon reflexes throughout both upper and lower extremities. She was able to walk independently with a forward‐leaning and wide‐based gait but was unable to run.

## 3 MATERIALS AND METHODS

### 3.1 Cytogenetic investigations

Comparative genomic hybridization (CGH) oligoarray using the platform (CytoChipTM ISCA 8x60K v2.0) was performed on DNA extracted from peripheral whole blood.

### 3.2 Biochemical testing

Plasma amino acids were quantitated after post‐column ninhydrin derivatization using a Biochrom 30+ amino acid analyzer (Biochrom Ltd, Cambridge, UK). Total homocysteine was measured using a heavy‐isotope dilution method following dithiothreitol reduction of disulfide bonds by liquid chromatography (LC)‐ tandem mass spectrometry (MS/MS) as previously described.^3^ Plasma SAM and SAH were measured using a heavy‐isotope dilution method after initial sample acidification, followed by neutralization and solid phase extraction by LC‐MS/MS as previously described.^4^ Urine creatine/guanidinoacetoacetate was measured at the Biochemical Genetics Laboratory at the Hospital for Sick Children (Toronto, Ontario, Canada), and urine purines and pyrimidines at the Laboratoire de génétique biochimique at the Centre Hospitalier Universitaire de Sherbrooke (CHUS; Sherbrooke, Québec, Canada).

### 3.3 Genetic analysis

Gene sequencing of the *AHCY* gene was performed at Baylor Miraca Genetics Laboratory (Houston, Texas). Gene sequencing of the *ADK* gene was performed at Centogene/Life Labs Laboratory (Rostock, Germany). XomeDxSlice analysis of the *ADA*, *GNMT*, *MAT1A*, *MTHFR*, and *SLC6A8* genes was performed at GeneDx Laboratory (Gaithersburg, Maryland).

Whole exome sequencing (WES) trio after slice was performed at GeneDx Laboratory (Gaithersburg, Maryland). Using genomic DNA extracted from peripheral lymphocytes, the Agilent Clinical Research Exome kit was used to target the exonic regions and flanking splice junctions of the genome. The targeted regions were sequenced simultaneously by massively parallel (NextGen) sequencing on an Illumina HiSeq sequencing system with 100 bp paired‐end reads. Bi‐directional sequence was assembled, aligned to reference gene sequences based on human genome build GRCh37/UCSC hg19, and analyzed for sequencing variants using a custom‐developed analysis tool (Xome Analyzer). The mean depth of coverage was 180× and quality threshold was 97.9%. Sanger sequencing was performed in the laboratory on the proband's and parents' DNA for variant confirmation.

## 4 RESULTS

### 4.1 Cytogenetic investigations

The array CGH was reported as normal female.

### 4.2 Biochemical testing

Plasma amino acid analysis at 23 months of age demonstrated an isolated 4.7‐fold elevation in plasma methionine at 212 μmol/L (Normal: 10‐45 μmol/L), and marginally elevated plasma total homocysteine level of 14.7 μmol/L (Normal: 3.1‐11.1 μmol/L). Plasma SAM had a 3.8‐fold elevation at 505 nmol/L (Normal: 86‐132 nmol/L), and plasma SAH had a 6.4‐fold elevation at 186 nmol/L (Normal: 9‐29 nmol/L). In addition, urine creatine was elevated at 1500 mmol/mol creatinine (normal: 14‐830 mmol/mol creatinine) and urine guanidinoacetoacetate was normal at 124 mmol/mol creatinine (normal: 5‐150 mmol/mol creatinine). She had normal urine adenosine levels. Creatine kinase (CK) was normal at 97 IU/L.

Plasma amino acids were repeated at 2 years, 2 months of age and she continued to have a 1.8‐fold elevation in plasma methionine (but within the context of other elevated amino acids suggestive of a nonfasted specimen), but with a normal plasma total homocysteine at 6.3 μmol/L. There remained a 2.3‐fold elevation of SAM, and an 8.1‐fold elevation in SAH. At 3 years of age, there was normalization of the plasma amino acids but persistence of elevated SAM (1.6‐fold) and SAH (2.4‐fold). At 4 years, 7 months of age, SAM was still marginally elevated, and SAH normalized. SAM‐to‐SAH ratios ranged from 1.31 to 3 between 23 months and 3 years of age and normalized by 4.7 years of age. Urine creatine and guanidinoacetoacetate levels normalized by 5 years of age as well.

#### 4.2.1 Molecular genetic testing

No pathogenic variants were found in the *ADK*, *ACHY*, *ADA*, *GNMT*, *MAT1A*, *MTHFR*, and *SLC6A8* genes. WES trio performed on the proband and both parents demonstrated a homozygous NM_018297.3(NGLY1):c.1405C>T (p.Arg469*) pathogenic variant in *NGLY1* in the proband, and heterozygosity for this *NGLY1* variant in the patient's mother and father. The variant had been reported in the homozygous state in an individual with unclassified epilepsy^5^ and was rare, with an allele frequency of 1.65 × 10^−5^ in ExAC, and no homozygotes reported.^6^ At the time of the writing of this manuscript, the variant was reported in ClinVar 3 times as pathogenic (one submission representing our patient). It is predicted to cause loss of normal protein function either through protein truncation or nonsense‐mediated RNA decay.

## 5 DISCUSSION

NGLY1‐CDDG presents with abnormal tear production, choreoathetosis, liver dysfunction, developmental delay, hypotonia, peripheral neuropathy, EEG abnormalities, and microcephaly (Table 1). Previously reported hepatic findings include elevated aminotransferases, fibrosis, neonatal jaundice, and intrahepatic cytoplasmic inclusions on biopsy.^2^, ^7^, ^8^ A publication from 2017 which prospectively phenotyped patients with NGLY1‐CDDG documented transient elevation of aminotransferases during the first 2 years of life, with normalization on average at age 4.^9^ Half of this cohort of 12 patients had normal abdominal ultrasound results, with abnormalities in the remainder including splenomegaly, steatosis, hepatomegaly, and coarse or inhomogeneous liver echotexture, and 3 of the 12 individuals had elevated fibroscan scores demonstrating possible liver fibrosis.

**Table 1:** Clinical features of the patients reported to date

Patients	Patient reference	Our patient	Trio 2	Patient 1	Patient 2	Patient 3	Patient 4	Patient 5	Patient 6 (Sib of 5)	Patient 7	Patient 8 (Sib of 7)	Patient 1	Patient 2 (Sib of 1)	Patient 1	Patient 2	Patients (n = 12)
	Article reference	Current article	Need et al	Enns et al								Caglayan et al		Heeley and Shiwani	Bosch et al	Lam et al
Genotype	Mutations	Missense c.1405C>T, exon 9	Nonsense mutation in exon 8	Frameshft c.1891del, exon 12	Frameshift c.1370dupG, exon 9	Stop gain c.1570C>	Nonsense c.1201A>T, exon 8	Nonsense c.1201A>T, exon 8	Nonsense c.1201A>T, exon 8	Nonsense c.1201A>T, exon 8	Nonsense c.1201A>T, exon 8	Frameshift c.1533_1536delTCAA	Frameshift c.1533_1536delTCAA	Stop gain c.347C > G, exon 3	Nonsense c.1201A > T, exon 8	13 mutations: 5 missense, 5 nonsense, 2 splice site, 1 frameshift
		Maternally inherited	Maternally inherited	Maternally inherited	Maternally inherited	Paternally inherited	Not reported	Matenally inherited	Matenally inherited	Not reported	Not reported	Matenally inherited	Matenally inherited	Maternally inherited	Maternally inherited	
		Missense c.1405C>T, exon 9	Frameshift variant in last exon	Nonsense c.1201A>T, exon 8	Frameshift c.1370dupG, exon 9	In frame deletion c.1205_1207del	Nonsense c.1201A>T, exon 8	Nonsense c.1201A>T, exon 8	Nonsense c.1201A>T, exon 8	Nonsense c.1201A>T, exon 8	Nonsense c.1201A>T, exon 8	Frameshift c.1533_1536delTCAA	Frameshift c.1533_1536delTCAA	Splice site abolished in exon 5, c.881 + 5G	Nonsense c.1201A > T, exon 8	
		Paternally inherited	Paternally inherited	Paternally inherited	Paternally inherited	Maternally inherited	Not reported	Paternally inherited	Paternally inherited	Not reported	Not reported	Paternally inherited	Paternally inherited	Paternally inherited	Paternally inherited	
Epidemiology	Age at last report	5	3	5	20	4	2	5	9.5 months	3	16	16	9	14	3	Range 2.5‐21.3 years
	Origin	Libya	European‐American	European, Puerto Rico	Italian	Caucasian	German	English, Ukraine, Finland	English, Ukraine, Finland	Caucasian	Caucasian	Caucasian	Caucasian	Caucasian		Caucasian
	Consanguinity	+		−	+	−	−	−	−	−	−	+	+			
	Gender	F	M	M	F	F	M	M	F	F	F	M	F	M	M	6 M; 6F
Pregnancy	Complications	Premature			IUGR, abnormal placenta, C/S	Non‐reassuring FHR, positive second trimester screen, C/S	IUGR, oligohydramnios, fetal distress, C/S	IUGR, fetal distress, positive second trimester screen, C/S	IUGR, positive second trimester screen, C/S, premature	Positive screen, planned C/S	IUGR, decreased fetal movement, bradycardia, C/S, required resuscitation	C/S due to nuchal cord		Vanishing twin		
	Weeks gestation (wk)	32			39	Term	38	36	35	Term	Term	Term		Term	37	Term (n = 10), 36 weeks (n = 1), 34 weeks (n = 1)
	Birth weight (gr)	1255												2722	2415	
Development	Global developmental delay	+	+	+	+	+	+	+	+	+	+	+	+	+	+	n = 12(/12)
	Intellectual disability	+			+							+	+	+		n = 9 (/12)
Neurological	Seizures	−	+	+	−	−	+	+	−	−	+	−	+	+	−	n = 7(/12)
	Movement disorder	+	+	+	+	+	+	+	+	+	+	−	+	+	+	n = 12(/12)
	Brain MRI abnormalities	+		+	−	+	+	+	+	−	+	−	−	−	+	Delayed myelination n = 3(/11), white matter lesions n = 2(/11), cerebral atrophy n = 6(/9), cerebellar atrophy n = 4(/11)
	Microcephaly	+		−	+	+	−	+	+	+	+		−	+	+	n = 4(/12)
	EEG findings	+		+	+	+	+	+	+	−	+	−	+	+		n = 8(/12)
	Peripheral neuropathy	Suspected		+	+			+				+	+	+	+	Axonal sensory polyneuropathy n = 8(/11), with demyelinating features n = 6(/11)
	Tremor	+			+											
	Hypotonia	+		+	+	+	+	+	+	+	+	+	+	+	+	
	Decreased DTRs	+		+	+	−	+	+	−	+	+	+	+	+		
	Other	Initial hypotonia, with later hypertonia in upper extremities		Cortical vision loss	Cortical vision loss	Staring spells		Regression age 4				Decreased pain sensitivity, decreased sweating. Thin cervical cord		Muscle atrophy	Delayed myelination	Low CSF protein, albumin, and CSF/serum albumin n = 12(/12)
Ophthalmological	Ocular apraxia	−		−	+	+	−	−	−	+	+	−	−	−		n = 0(/11)
	Hypo‐ or alacrima	+	+	+	+	+	+	+	−	+	+	+	+	+	+	n = 11(/11)
	Other	Myopia, astigmatism, esotropia, retinal hemorrage (infant), subsequent normal dilated fundus exam				Strabismus	Strabismus, Bilateral ptosis			Strabismus	Strabismus	Ptosis, strabismus		Lacrimal duct stenosis, strabismus, ptosis, blepharitis	Strabismus	Ptosis n = 5(/11), lagophthalmos n = 9(/11), nystagmus n = 2(/11), strabismus n = 5(/11), optic atrophy n = 6(/11)
Audiological	Hearing impairment	−		−	−	+	+	−			+	−	−	−	−	Delayed or absent auditory brainstem response n = 9(/11)
Cardiac	QTcB prolonged	−														n = 2(/12)
Sleep	Sleep abnormalities	Bruxism, sleep talking														Obstructive sleep apnea n = 2(/9), central sleep apnea n = 1(/9), combined n = 2(/9), frequent periodic limb movements n = 5(/9)
Feeding	Poor feeding	+				+						+	+	Poor growth	+	Oral motor deficits n = 10(/11), premature spillage and pharyngeal swallow response delay n = 11(/11)
Musculoskeletal	Delayed bone age	+														n = 8(/11)
	Low bone density/recurrent fractures	+										+	−	+		n = 9(/9)
	Joint hypermobility or dislocations	+														Dislocation or subluxation of hips/shoulder n = 3(/11)
	Sclerosis of phalanges or tarsal bones	−														n = 2(/11)
	Scoliosis	−		−	+	−	+	−	−	−	+	+	−	+		n = 6(/11)
	Other							Flexion contractures knees			Talipes equinovarus	Contractures at ankles			Extension restriction of knee	Coxa valga n = 11(/11), growth arrest lines or metaphyseal banding n = 4(/11)
Gastrointestinal	Constipation	+		+	+	+	+	+	−	+	+			+		n = 10(/12)
	Abnormal liver function/elevated transaminases	+	+	+	+	+	+	+		+	−	+	−	+		Early elevations with later normalization n = 8(/8)
	Cholesterol and TGs low	−														+
	Liver fibrosis	+		+	−	−	+	−	−			−	−	+		n = 3(/12)
	Liver storage or vacuolization			+	+	+	−	+	+					+		n = 0(/3)
	Other	Neonatal cholestasis, meconium plug		Neonatal jaundice		Neonatal jaundice	Neonatal jandice		Neonatal jaundice	Neonatal jaundice	GERD, anal stenosis requiring dilatation	Hepatomegaly		Neonatal jaundice		Abnormal abdominal ultrasound (steatosis, splenomegaly, coarse/inhomogenous liver texture, hepatomegaly) n = 6(/12), hepatocellular carcinoma n = 1(/12)
Hematologic	Coagulation studies abnormal	Transient thrombocytopenia				Transient thrombocytopenia										Low protein C activity n = 6(/12), factor II activity n = 1(/12), factor IX n = 2(/12), factor XI n = 2(/12), fibrinogen n = 5(/12)
Immunologic	Abnormal antibody titres	−														Elevated rubella/rubeola antibody titres after MMR n = 7(/11)
Lab and biochemical findings	Liver biopsy		Amorphous substance											Cirrhosis, macrosteatosis, lipid accumulation with dilated ER		No storage material identified (n = 3)
	Muscle biopsy													Minor variation in muscle fiber size, normal mitochondrial stains		
	TIEF	normal	normal											−		
	N‐glycan analysis		normal													normal n = 9(/9)
	*O*‐glycan analysis															normal n = 8(/8)
	Lactic acidosis	−		−	+	+	+	−		+		−	−	−		n = 5(/12)
	Urine MPS															Elevated n = 4(/8)
	Other													Hypocholesteolemia		
Dysmorphic features	Dysmorphic facies	+		−	−	−	−	+	+	+	+		+	+	+	+
	Other	Deep set eyes, short palpebral fissues, synophrys with full, arched eyebrows. Bilateral 5th finger clinodactyly, fetal pads on all fingers		Small hands/ft	Distal tapering hands and feet							Unilateral cryptorchidism	Hypertelorism, single right palmar crease	Myopathic face, hypertelorism, wide mouth	Metopic ridge, medial flaring eyebrows, long upslanting palpebral fissures, small mouth and ears, tapering fingers, bilateral clinodactyly IV and V toes, hypoplastic toenails, small penis, cryptorchidism	Small feet n = 12(/12)
Other remarks		Patent ductus arteriosus and atrial septal defect, resolved				AFP 1.63 MoM, uE3 0.26 MoM, hCG 0.54 MoM, inhibin 1.02 MoM		AFP 1.97 MoM, uE3 0.24 MoM, hCG 0.48 MoM Deceased age 5, viral illness/ prolonged seizure	AFP 0.87 MoM, uE3 0.31 MoM, hCG 0.57 MoM Deceased age 9.5 months in sleep, cause unknown			Deceased age 16 ‐ respiratory difficulties and recurrent infections		Elevated LFTs first noted at 14 days, mild (ALT<201, AST <236). Complete normalization of AST age 8 and ALT age 13. No associated hepatomegaly		Six individuals (patients 2, 3, 4, 9, 11, and 12) were included in previous clinical publications

Abbreviations: AFP, alpha feto‐protein; ALT, alanine aminotransferase; AST, aspartate aminotransferase; C/S, Caeserian section; CSF, cerebrospinal fluid; DTRs, deep tendon reflexes; EEG, electroencephalogram; ER, endoplasmic reticulum; F, female; FHR, fetal heart rate; GERD, gastroesophageal reflux; hCG, human chorionic gonadotropin; IUGR, intrauterine growth restriction; LFT, liver function test; M, male; MMR = measles/mumps/rubella; MoM, multiples of the median; MPS, mucopolysaccharides; MRI, magnetic resonance imaging; QTcB, QT interval corrected for heart rate; Sib, sibling; TG, triglycerides; TIEF, transferrin isoelectric focusing; uE3, unconjugated estradiol.

The mechanism by which loss of NGLY1 activity leads to the clinical phenotype is not well understood. Loss‐of‐function mutations in *NGLY1* appears to lead to accumulation of misfolded proteins, which may interfere with cellular functions. The enzyme is known to play a key role in quality control of misfolded N‐glycosylated proteins, as cleavage of the attached N‐glycans directly precedes proteasomal degradation.^10^ Regulation of proteolysis through de‐N‐glycosylation of Nrf1, a transcription factor that upregulates proteasome subunit gene expression, has also been demonstrated in vitro, with a clear role between NGLY1 activity and regulation of proteostasis.^11^ In the absence of NGLY1, cytosolic endo‐β‐N‐acetylglucosaminidase (ENGase) acts on misfolded glycoproteins to generate N‐GlcNAc proteins, which are hypothesized to cause toxic effects on cells through protein aggregation and/or impairment of intracellular signalling pathways.^12^, ^13^ Patients who have NGLY1 deficiency have been described to have mitochondrial dysfunction as observed on muscle and liver biopsy and in vitro, potentially implicating a role for NGLY1 in the respiratory chain.^1^, ^2^, ^14^

**Figure 2:**
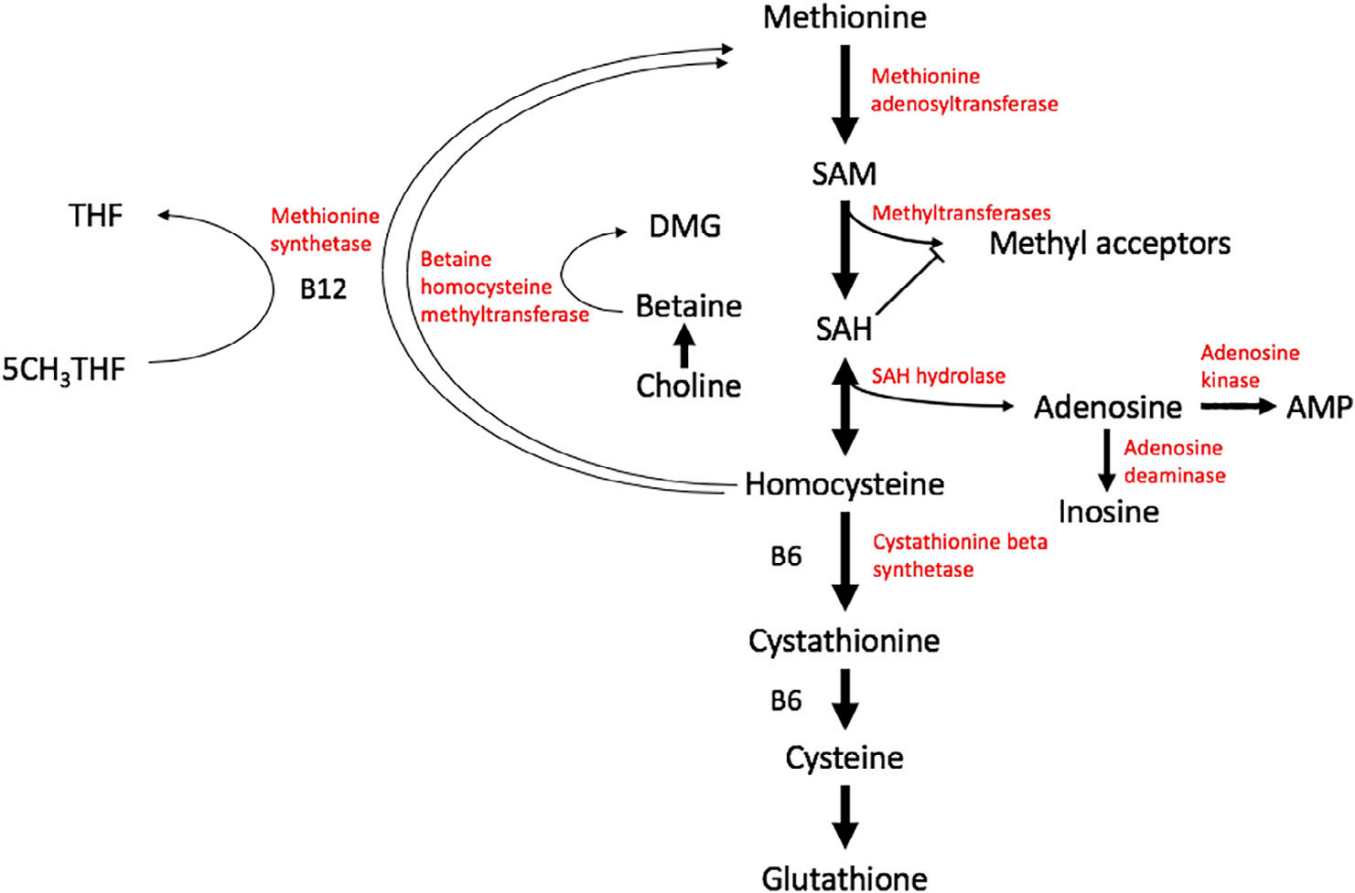
Methylation and remethylation pathway demonstrating SAM and SAH as key intermediates. AMP, adenosine monophosphate; DMG, dimethylglycine; SAM, *S*‐adenosylmethionine; SAH, *S*‐adenosylhomocysteine; THF, tetrahydrofolate. Adapted from Melnyk S, Pogribna M, Pogribny IP, Yi P, James SJ. Measurement of plasma and intracellular SAdenosylmethionine and *S*‐adenosylhomocysteine utilizing coulometric electrochemical detection: alterations with plasma homocysteine and pyridoxal 5’‐phosphate concentrations. *Clin Chem*. 2000;272:265‐272

Our patient demonstrated elevations in methionine, SAM, and SAH, and mild elevation in homocysteine. To our knowledge, this is the first report of this biochemical profile in patients with NGLY1‐CDDG. Methionine is activated in an ATP‐dependent reaction to form SAM, which plays a major role in methyl donation during biosynthetic reactions. SAH is formed from the demethylation of SAM following donation of the methyl group to an acceptor; the adenosyl group is subsequently removed to form homocysteine (Figure 2).

With respect to the potential mechanism that may underlie the SAM and SAH elevations in our patient, we considered several possibilities. Elevations in homocysteine levels due to low dietary folate or B12 may present with low SAM:SAH ratios and elevated SAH, but typically not with elevated SAM.^15^ Folate was not checked, but vitamin B12 and methylmalonic levels were normal, suggesting that this is a less likely explanation for our patient. While liver disease in patients with NGLY1‐CDDG has been characterized in multiple reports, hepatic SAM metabolism in patients with chronic liver disease typically shows patterns of low SAM due to SAM depletion and/or reduced synthesis, rather than elevation.^16^ Therefore, this is an unlikely explanation as well. Very elevated methionine levels (up to 26‐fold) and elevated SAM have been described in patients with hepatic mitochondrial DNA (mtDNA) depletion syndromes, but were not accompanied by elevations in SAH.^17^ It is also possible that muscle involvement may be contributing to our patient's presentation, however, her creatine kinase level has been normal to date.

Genetic testing in our patient for enzymes involved in methylation was unrevealing. Multiple levels of control over methylation enzymes have been described, including oxidative stress, metabolites from the same or related pathways, and hormones and nutrients.^18^ We suggest that dysregulation of one or more enzymes involved in methylation may be the cause for our patient's presentation, through one or more of the pathways impacted by loss of NGLY1. The elevations in methionine, homocysteine, SAM, and SAH improved over time, suggesting that the implicated enzymatic dysregulation is reversible. She also had low SAM:SAH ratios from 23 months to 3 years of age, which may suggest reduced methylation capacity.^19^ While our findings are limited to a single case, it may be informative to explore whether SAM and SAH are also dysregulated in other patients with NGLY1‐CDDG, and whether a pattern of methylation differences can be detected in this group of patients.

## 6 CONCLUSION

In summary, we report a new case of NGLY1‐CDDG with transient elevations in methionine and homocysteine, as well as SAM and SAH, which are involved in single‐carbon metabolism. Our goal is to expand the reported biochemical phenotype of patients with NGLY1‐CDDG, with possible future research avenues including evaluation of the regulation of enzymes involved in methylation through one or more of the pathways impacted by loss of NGLY1.

## AUTHOR CONTRIBUTIONS

C. A. C.––Contributed significantly to writing the manuscript, saw the patient, and consented the family for publication. S. R. M.––Gastroenterologist following the patient, critically reviewed and edited the manuscript. D. S. S. ––Biochemical Geneticist essential in the completion, analysis, and interpretation of biochemical laboratory results, critically reviewed and edited the manuscript. W. A.‐H.––Primary provider for the patient and directed diagnostic laboratory investigations, confirmation of diagnosis, follow‐up and management of the patient. Responsible for planning, conducting, and reporting the case. Contributed significantly to the manuscript, and critically reviewed and edited the manuscript.

## COMPLIANCE WITH ETHICS GUIDELINES

Caitlin A. Chang, Xing‐Chang Wei, Steven R. Martin, David S. Sinasac, and Walla Al‐Hertani declare that they have no conflict of interest. No funding sources were required for this work. Publication of an unreported clinical case does not require REB review as per second edition of the Tri‐Council Policy Statement: Ethical Conduct for Research Involving Humans (TCPS 2). All procedures followed were in accordance with the ethical standards of the responsible committee on human experimentation (institutional and national) and with the Helsinki declaration of 1975, as revised in 2000 (5). Written informed consent was obtained from the patient's parents prior to the case publication, for being included in the study, and is available for review upon request. This article does not contain any studies with animal subjects performed by any of the authors.

## PATIENT CONSENT

The patient was seen at the Alberta Children's Hospital in Calgary, Alberta, Canada, and written parental consent was obtained for publication.
